# Crystal structure of a *Pseudomonas* malonate decarboxylase holoenzyme hetero-tetramer

**DOI:** 10.1038/s41467-017-00233-z

**Published:** 2017-07-31

**Authors:** Riyaz Maderbocus, Blanche L. Fields, Keith Hamilton, Shukun Luo, Timothy H. Tran, Lars E. P. Dietrich, Liang Tong

**Affiliations:** Department of Biological Sciences, Columbia University, New York, NY 10027 USA

## Abstract

*Pseudomonas* species and other aerobic bacteria have a biotin-independent malonate decarboxylase that is crucial for their utilization of malonate as the sole carbon and energy source. The malonate decarboxylase holoenzyme contains four subunits, having an acyl-carrier protein (MdcC subunit) with a distinct prosthetic group, as well as decarboxylase (MdcD–MdcE) and acyl-carrier protein transferase (MdcA) catalytic activities. Here we report the crystal structure of a *Pseudomonas* malonate decarboxylase hetero-tetramer, as well as biochemical and functional studies based on the structural information. We observe a malonate molecule in the active site of MdcA and we also determine the structure of malonate decarboxylase with CoA in the active site of MdcD–MdcE. Both structures provide molecular insights into malonate decarboxylase catalysis. Mutations in the hetero-tetramer interface can abolish holoenzyme formation. Mutations in the hetero-tetramer interface and the active sites can abolish *Pseudomonas aeruginosa* growth in a defined medium with malonate as the sole carbon source.

## Abstract (web summary)

Some aerobic bacteria contain a biotin-independent malonate decarboxylase (MDC), which allows them to use malonate as the sole carbon source. Here, the authors present the crystal structure of a *Pseudomonas* MDC and give insights into its catalytic mechanism and function.

## Introduction

Malonate can be used as the sole carbon and energy source by various bacterial organisms, such as *Klebsiella pneumoniae*, *Acinetobacter calcoaceticus*, *Pseudomonas putida*, and *Malonomonas rubra* ^1–3^. Decarboxylation of malonate to produce acetate and CO_2_ is the crucial activity for its utilization. In *M. rubra* and other anaerobic organisms, a membrane-bound, biotin-dependent malonate decarboxylase (MAD) system couples malonate decarboxylation to Na^+^ transport across the cell membrane, and this system is related to oxaloacetate decarboxylase and other Na^+^-translocating decarboxylases^4, 5^. In *K. pneumoniae*, *A. calcoaceticus*, *P. putida*, *Pseudomonas aeruginosa*, and other aerobic organisms, the decarboxylation is mediated by a cytosolic, biotin-independent malonate decarboxylase (MDC) system. Both systems contain an acyl-carrier protein (ACP) to activate malonate for decarboxylation, and therefore they are distinct from the malonyl-CoA decarboxylase (MCD) enzyme^6, 7^.

In *K. pneumoniae*, the MDC system is encoded by an operon with nine genes, *mdcABCDEFGHR* ^8^. MdcR is the transcription factor regulating the expression of this operon, and MdcF is a malonate transporter in the cell membrane. The MDC operons in *A. calcoaceticus* ^2, 9–11^, *P. putida* ^12^, and *P. aeruginosa* are organized somewhat differently, but they contain the same essential components as the operon in *K. pneumoniae*. For example, the operon contains *mdcABCDEGHLM* in *P. putida* ^12^ and *P. aeruginosa*, with MdcL–MdcM comprising the malonate transporter.

**Figure 1:**
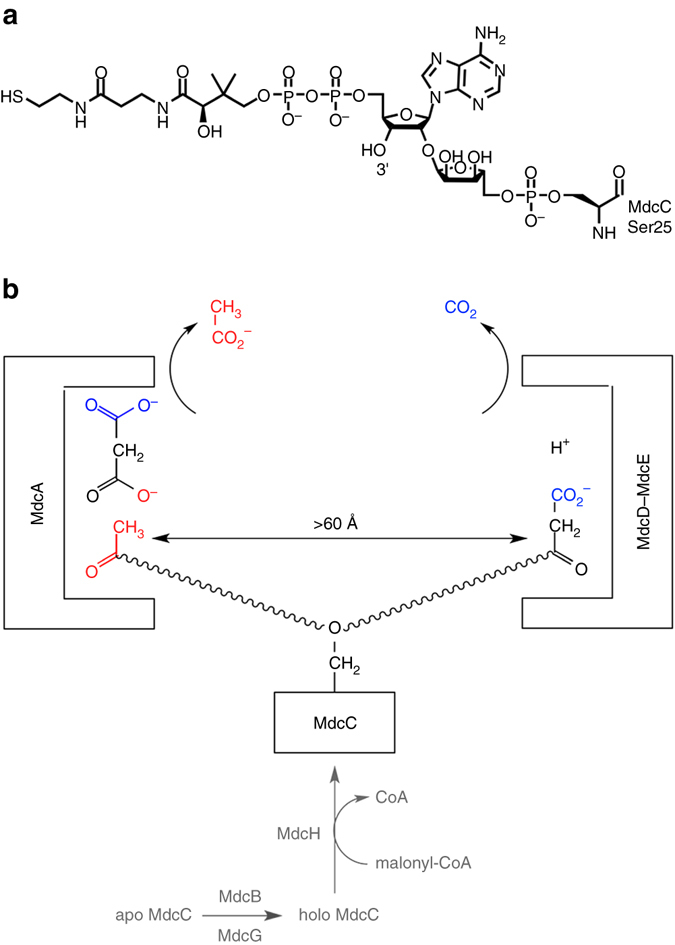
Reactions catalyzed by the malonate decarboxylase (MDC) system. **a** Chemical structure of the 2′-(5′′-phosphoribosyl)-3′-dephospho-CoA prosthetic group for the acyl-carrier protein (MdcC) in malonate decarboxylase. **b** Malonate decarboxylase contains two distinct active sites. MdcA is an acetyl-ACP:malonate ACP transferase and converts free malonate to malonyl-ACP, which is then decarboxylated by MdcD–MdcE. The prosthetic group of the MdcC is indicated with the wavy lines. MdcB, MdcG and MdcH are involved in activating the ACP for catalysis (shown in *gray*)

MdcA, MdcC, MdcD, and MdcE form the 140 kD MDC holoenzyme, with 1:1:1:1 stoichiometry among the four subunits. MdcC (10 kD, Supplementary Fig. 1) is the ACP, and its Ser25 side chain carries the 2′-(5′′-phosphoribosyl)-3′-dephospho-CoA prosthetic group (Fig. 1a), a post-translational modification catalyzed by MdcB and MdcG (Fig. 1b)^13, 14^. The free thiol on ACP is activated for catalysis by reacting with malonyl-CoA, which is catalyzed by MdcH, a malonyl-CoA:ACP transacylase. MdcH is loosely associated with the MDC holoenzyme and can copurify with it in sub-stoichiometric amounts^15, 16^.

MdcA (60 kD, Supplementary Fig. 1) is an acetyl-ACP:malonate ACP transferase and catalyzes the first step in the cyclic reaction of malonate decarboxylation, converting free malonate to malonyl-ACP (Fig. 1b)^3^. MdcA belongs to family II of the large collection of ACP transferases, while family I enzymes transfer CoA between various organic acids^17^. The two families of enzymes share sequence homology, but their catalytic mechanisms are different. Family I enzymes operate with a ping-pong mechanism, through the formation of a covalent adduct with an acidic side chain (most often Glu). In contrast, family II enzymes (ACP transferases) operate through the formation of a ternary complex with both substrates, and do not require a covalent adduct as the intermediate.

The malonyl-ACP is decarboxylated in the second step of the reaction to regenerate acetyl-ACP, which is catalyzed by MdcD (30 kD, Supplementary Fig. 2) and MdcE (28 kD, Supplementary Fig. 2). The reaction consumes a proton and produces CO_2_ rather than bicarbonate from the decarboxylation (Fig. 1b)^18^. MdcD has 35% sequence identity with the β subunit of the carboxyltransferase (CT) component of *Escherichia coli* acetyl-CoA carboxylase (ACC), suggesting that MdcD–MdcE could share a similar mechanism as CT. The sequence homology between MdcE and the α subunit of *E. coli* CT is much lower.

While proteins of the MDC operon from diverse species have been studied extensively at the biochemical level, no structural information is available for them. We report here the crystal structures of the MdcD–MdcE complex as well as an MDC MdcA–MdcC–MdcD–MdcE hetero-tetramer from *Pseudomonas* species, with the MdcA, MdcD, and MdcE subunits from *P. aeruginosa* and the MdcC subunit from *Pseudomonas fluorescens*. MdcA has structural similarity to CoA transferases while MdcD–MdcE is similar to the CT component of ACC. The structure reveals extensive interactions at the subunit interfaces in the hetero-tetramer. We have also determined the binding modes of malonate in the active site of MdcA and CoA in the active site of MdcD–MdcE. These structures provide molecular insights into MDC catalysis. Mutations in the hetero-tetramer interface can abolish holoenzyme formation, and our functional studies showed that mutations in the hetero-tetramer interface as well as in the holoenzyme active sites can abolish *P*. *aeruginosa* growth in a defined medium with malonate as the sole carbon source.

## Results

### Overall structure of MdcD–MdcE complex

We determined the crystal structure of the *P. aeruginosa* MdcD–MdcE complex at 1.86 Å resolution by the selenomethionyl single-wavelength anomalous diffraction method. The current atomic model has excellent agreement with the crystallographic data and the expected bond lengths, bond angles, and other geometric parameters (Table 1). A total of 98.5% of the residues are in the favored region of the Ramachandran plot, and no residues are in the disallowed region. The two copies of the MdcD–MdcE complex in the asymmetric unit have essentially the same conformation, with overall rms distance of 0.4 Å between their equivalent Cα atoms. Several segments of both subunits are disordered, especially in the second copy of the MdcD–MdcE complex.

**Table 1:** Crystallographic data collection and refinement statistics

Structure	MdcD–MdcE hetero-dimer	MDC hetero-tetramer	MDC hetero-tetramer
		Malonate complex	CoA complex
*Data collection*			
Space group	*P*1	*P*1	*P*2_1_
Cell dimensions			
*a*, *b*, *c* (Å)	56.3, 60.1, 91.3	93.5, 161.4, 102.7	98.7, 163.6, 100.4
*α*, *β*, *γ* (°)	89.9, 88.8, 68.8	90.8, 93.8, 90.1	90, 94.0, 90
Resolution (Å)^a^	46–1.86 (1.92–1.86)	50–2.2 (2.28–2.2)	50–3.0 (3.18–3.0)
*R*_merge_ (%)	6.2 (38.6)	7.2 (45.7)	10.0 (67.9)
CC_1/2_	–	–	99.4 (72.1)
*I*/*σI*	12.7 (2.5)	9.6 (1.6)	12.0 (1.7)
Completeness	96.0 (92.6)	97.4 (95.3)	98.8 (97.9)
Redundancy	3.1 (3.0)	1.9 (1.8)	3.2 (3.3)
*Refinement*			
Resolution (Å)	46–1.86 (1.90–1.86)	50–2.2 (2.23–2.2)	50–3.0 (3.04–3.0)
No. reflections	88,297	294,062	63,159
*R*_work_/*R*_free_ (%)	18.1 (22.6)/20.8 (25.0)	18.6 (27.3)/23.2 (33.1)	17.3 (32.9)/23.3 (39.0)
No. atoms			
Protein	7474	35,976	18,104
Ligand/ion	0	28	97
Water	216	1459	0
B-factors			
Protein	23.9	36.2	64.6
Ligand/ion	–	27.8	67.1
Water	24.8	34.8	–
rms deviations			
Bond lengths (Å)	0.008	0.009	0.010
Bond angles (°)	1.0	1.2	1.5
PDB entry code	5VIP	5VIT	5VJ1

^a^The numbers in parentheses are for the highest resolution shell

**Figure 2:**
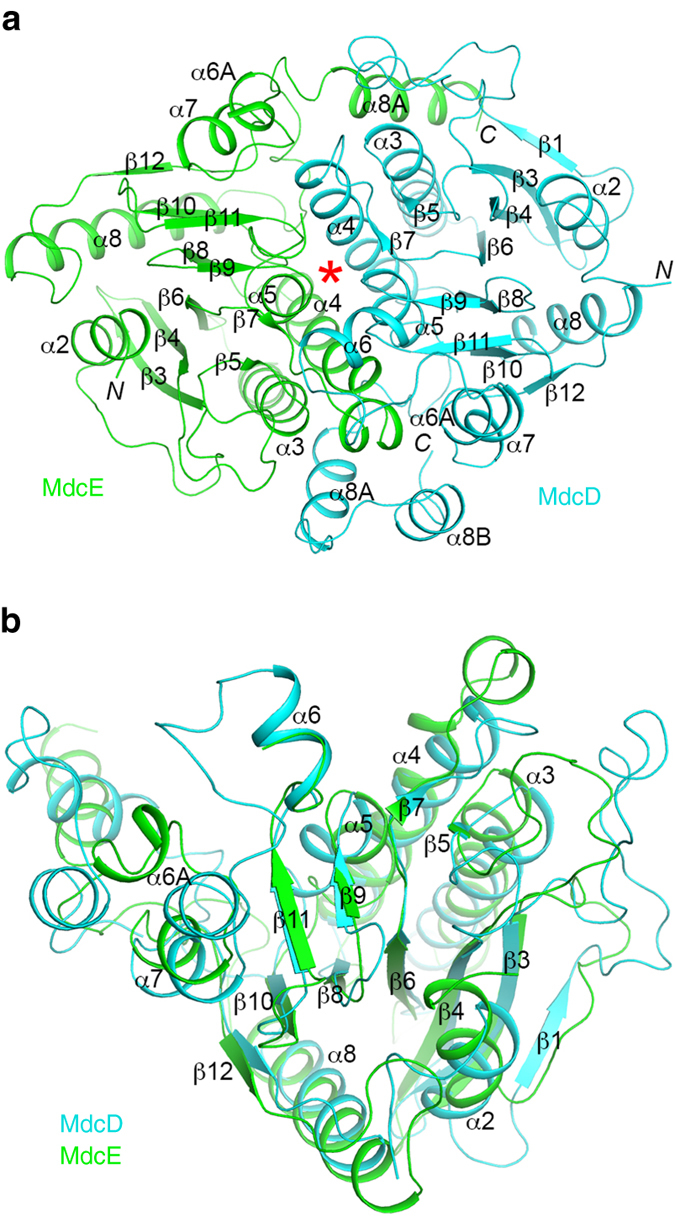
Structure of the MdcD–MdcE complex. **a** Schematic drawing of the structure of the *P. aeruginosa* MdcD–MdcE complex. MdcD is shown in *cyan*, and MdcE in *green*. The secondary structure elements are named following the convention of yeast ACC CT domain. The active site is indicated with the *red asterisk*. **b** Overlay of the structures of MdcD (*cyan*) and MdcE (*green*). The β-strands in the β–β–α superhelix are mostly superposed. All structure figures were produced with the program PyMOL (www.pymol.org)

The structures of the MdcD and MdcE subunits have the crotonase fold, with a β–β–α motif that is repeated four times in its core (β–β–α superhelix) (Fig. 2a). The first β-strand from each repeat (β4, β6, β8, and β10) forms a large, parallel β-sheet, while the second β-strand of each repeat (β5, β7, β9, and β11) forms a smaller β-sheet that is perpendicular to that formed by the first strands (Fig. 2a). (The secondary structure elements are named following the system for the yeast ACC CT domain^19^.) The crossover helices (α3, α4, and α5) are located on one side of the large β-sheet.

The structures of MdcD and MdcE can be superposed to give a rms distance of 1.6 Å for 148 equivalent Cα atoms located within 3 Å of each other, mostly aligning residues in the β–β–α superhelix (Fig. 2b). The sequence identity of the equivalent residues is 22%, and that between all the residues of the two subunits is much lower (a Blast search with MdcD sequence failed to detect MdcE).

The MdcD–MdcE complex is formed by juxtaposing the crossover helices of the two subunits (Fig. 2a), burying ~3200 Å^2^ of the surface area of each subunit, and residues in the interface are indicated in Supplementary Fig. 2. This places their two small β-sheets into proximity and in the same plane, with the C-terminal ends of their β-strands pointed at each other (Fig. 2a). The active site of the MdcD–MdcE complex is located at this interface (see below). The two subunits are related by a rotation of 173° in this complex, suggesting pseudo two-fold symmetry between them.

The *A. calcoaceticus* MdcD–MdcE complex was reported to be a 2:2 hetero-tetramer in solution, and the MdcD subunit itself could also dimerize^10^. In contrast, the *P. aeruginosa* MdcD–MdcE complex is a 1:1 hetero-dimer in solution based on our gel filtration data, and a 2:2 hetero-tetramer was not observed in the crystal either.

**Figure 3:**
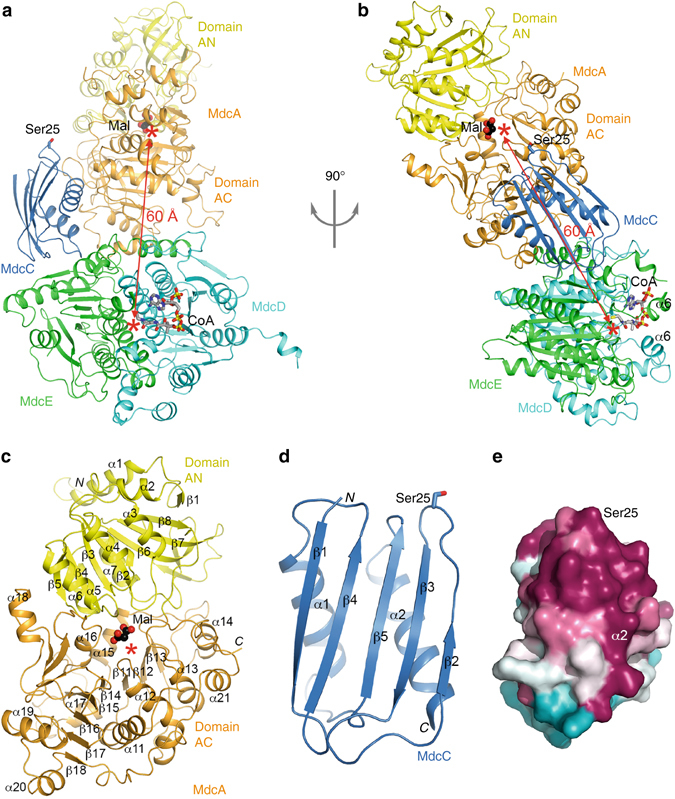
Structure of the MDC hetero-tetramer. **a** Schematic drawing of the structure of the MDC hetero-tetramer. The two domains of MdcA, AN and AC, are shown in *yellow* and *orange*, respectively. MdcC is shown in *light blue*, MdcD in *cyan*, and MdcE in *green*. The side chain of Ser25 in MdcC, the site of attachment of the prosthetic group, is shown as a stick model. A malonate bound in the active site of MdcA is shown as a sphere model (*black*) and labeled Mal. The bound position of CoA is also indicated (*gray*). The two active sites are indicated with the *red asterisks*, and the direct distance between them indicated with the *red arrow*. **b** Structure of the MDC hetero-tetramer, after 90° rotation around the vertical axis from **a**. **c** Structure of MdcA. **d** Structure of MdcC. **e** Molecular surface of MdcC colored based on sequence conservation^50^. *Purple*: highly conserved, cyan: poorly conserved. The view is related to that of **d** after a 90° rotation around the vertical axis

The structures of MdcD and MdcE share strong homology with other proteins that have the crotonase fold, especially the CT components of acyl-CoA carboxylases. For example, the structure of MdcD can be superposed with that of the β subunit of *S. aureus* ACC CT^20^ to give a rms distance of 1.5 Å for 178 equivalent Cα atoms (Supplementary Fig. 3). Moreover, this superposition brings MdcE into general superposition with that of the α subunit of *S. aureus* CT, indicating a conserved organization of the two enzymes. One difference between MdcD–MdcE and CT is that the latter is an α_2_β_2_ hetero-tetramer. The structural overlay with the *S. aureus* CT indicates that the C-terminal helices (α8A and α8B) of MdcD clash with the second copy of the *S. aureus* CT (Supplementary Fig. 3) and therefore the MdcD–MdcE complex is incompatible with the CT hetero-tetramer, which may explain why *Pseudomonas* MdcD–MdcE is a 1:1 complex. On the other hand, the sequence conservation of these two C-terminal helices is weaker compared to the rest of the MdcD protein (Supplementary Fig. 2), which might allow *A. calcoaceticus* MdcD to assume a different conformation here to produce a 2:2 MdcD–MdcE complex.

### Overall structure of the MDC hetero-tetramer

We next determined the crystal structure at 2.2 Å resolution of the MDC hetero-tetramer, with the MdcA, MdcD, and MdcE subunits from *P. aeruginosa* and the MdcC subunit from *P. fluorescens*. We were able to obtain crystals of the *P. aeruginosa* MDC hetero-tetramer, but the diffraction quality was very poor. The hybrid hetero-tetramer with the *P. fluorescens* MdcC, which has 76% identity with *P. aeruginosa* MdcC (Supplementary Fig. 1), produced much better crystals. MdcC did not carry the prosthetic group, as we were not able to express the modifying enzymes in *E. coli* (Fig. 1b). A malonate molecule from the crystallization solution is bound in the MdcA active site (Fig. 3a, see below), so this structure is actually the complex with malonate. The current atomic model has good agreement with the crystallographic data and the expected bond lengths, bond angles and other geometric parameters (Table 1, Supplementary Fig. 4). The four copies of the hetero-tetramers in the asymmetric unit have similar conformation, with overall rms distances of 0.2–0.4 Å between any pair of their equivalent Cα atoms.

**Figure 4:**
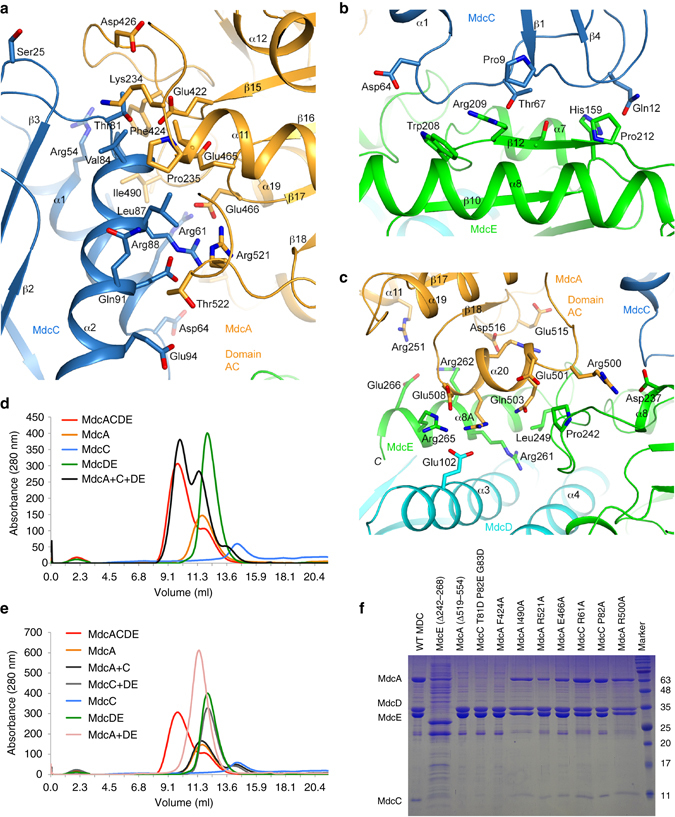
Interactions among the subunits in the MDC hetero-tetramer. **a** Interactions between MdcA (domain AC, *orange*) and MdcC (*light blue*) in MDC. **b** Interactions between MdcC (*light blue*) and MdcE (*green*) in MDC. **c** Interactions between MdcA (domain AC, *orange*) and MdcE (*green*) in MDC. MdcD (*cyan*) makes a small contribution to this interface. **d** Gel filtration profiles showing that the MDC hetero-tetramer can be formed by mixing its subunits. MdcACDE: the hetero-tetramer purified from co-expressing the four subunits; MdcA+C+DE: mixture of purified MdcA, MdcC, and MdcD–MdcE subunits. **e** Gel filtration profiles showing that the sub-complexes of MDC are not stable, with the exception of MdcD–MdcE. **f** Nickel pull-down experiments showing the effects of mutations in the MDC interface on hetero-tetramer formation. Only MdcE carries a His tag. The deletion mutations abolished the complex, as no MdcA nor MdcC was pulled down, while most of the single-site mutations had essentially no effect

By soaking crystals of the hetero-tetramer with malonyl-CoA, we obtained the structure of MDC in complex with CoA at 3.0 Å resolution (Table 1; the malonate group was likely hydrolyzed during the soaking). The two hetero-tetramers in the asymmetric unit of this crystal have essentially the same conformation, with rms distance of 0.1 Å for their equivalent Cα atoms. The overall structure of this hetero-tetramer is similar to that of the malonate complex, with rms distance of ~ 0.4 Å for their equivalent Cα atoms. The electron density in the active site of MdcA is smaller than malonate in this structure, and a chloride ion is modeled in one of the active sites.

The MDC hetero-tetramer has an elongated shape overall, with dimensions of ~40 Å × 60 Å × 130 Å (Fig. 3a, b). The MdcD–MdcE hetero-dimer forms the base of the structure, and the structure of this complex in the hetero-tetramer is essentially the same as that of the hetero-dimer alone, with rms distance of 0.4 Å. The MdcA subunit contacts the side of the MdcD–MdcE complex. The MdcC subunit contacts both MdcA and MdcE, and its Ser25 residue is pointed toward the active site of MdcA (see below).

A dimer of the MDC hetero-tetramer is observed in the crystal, mediated solely through the MdcA subunit (Supplementary Fig. 4). The buried surface area of this dimer is rather extensive, ~4200 Å^2^ for each MdcA subunit. However, earlier reports^1, 2^ as well as our gel filtration data on the MDC holoenzyme consistently show that it is a monomer in solution, with molecular weight of about 140 kD. Therefore, the observed dimer is probably induced by the high concentration of the enzyme during crystallization. Nonetheless, the large buried surface area suggests that this surface of MdcA could have a function for MDC, and further studies will be necessary for its characterization.

### The structure of MdcA

The structure of MdcA can be divided into two domains, formed by its N-terminal (1–223, domain AN) and C-terminal (224–554, domain AC) residues (Fig. 3a, c). Both domains have a Rossmann fold core. Domain AN has a central, eight-stranded (β1–β8) fully parallel β-sheet, while domain AC has the six-stranded (β11–β16; secondary structure elements in this domain are numbered from 11 upwards) β-sheet of the Rossmann fold, as well as two additional anti-parallel β-strands beyond β16. The planes of the β-sheets in the two domains are nearly perpendicular to each other, and the C-terminal ends of their β-strands are pointed toward each other. The active site of the subunit is located at this interface between the two domains, where a malonate molecule is bound (Fig. 3c, see below). Domain AC also contains a long C-terminal extension (residues 515–554, after strand β18), which wraps around the helices that cover one face of its central β-sheet.

The structures of the AN and AC domains can be superposed to give a rms distance of 1.7 Å for 106 equivalent Cα atoms located within 3 Å of each other (Supplementary Fig. 5). This accounts for less than half of the residues in each domain, and the sequence identity of the equivalent residues is only 11%. The two domains are related by a rotation of 179°, suggesting pseudo two-fold symmetry between them.

The overall fold of MdcA is similar to that of family I CoA transferases and family II ACP transferases, although there are also substantial structural differences. In addition, the relative positions of the two domains also show differences among these enzymes. Based on a search in the PDB with the program DaliLite^21^, the closest homologs for domain AN include family I CoA transferases such as succinyl-CoA:3-ketoacid CoA transferase (SCOT)^22–24^ (Supplementary Table 1, Supplementary Fig. 5), acetyl-CoA transferase^25^, acyl-CoA transferase YdiF^26^, gluconate-CoA transferase^27^, and succinyl-CoA:acetate CoA transferase^28^, and family II ACP transferase citrate lyase α subunit (citrate-ACP transferase). With domain AN superposed onto its equivalent domain in SCOT, the orientation of domain AC differs from its equivalent in SCOT by 15° (Supplementary Fig. 5).

The closest homologs for domain AC include family I CoA transferases 4-hydroxybutyrate CoA transferase^29^ (Supplementary Table 1), succinyl-CoA:acetate CoA transferase^28^, and SCOT, and family II ACP transferase citrate lyase α subunit (Supplementary Fig. 5). With domain AC superposed onto its equivalent in citrate lyase α subunit, the orientation of domain AN differs from its equivalent by 7° (Supplementary Fig. 5). This structure of the citrate lyase α subunit is in complex with citrate, which is also bound at the domain interface, although not as deep as malonate in MDC (Supplementary Fig. 5).

### The structure of MdcC

The structure of MdcC contains a five-stranded (β1–β5) mixed β-sheet, with two helices on one face (Fig. 3d). Ser25 is located at the tip of a long loop connecting anti-parallel strands β2 and β3, and is fully accessible for covalent modification. It is surrounded by a patch of conserved residues among MdcC homologs (Fig. 3e), which may be important for its role in MDC catalysis, as well as for recognition by MdcG for post-translational modification (Fig. 1b).

MdcC does not have a close structural homolog in the PDB. The top hits from a DaliLite search, with *Z* scores of ~6, include domain IV of elongation factor G^30, 31^ (Supplementary Fig. 5), the catalytic domain of *E. coli* Lon protease^32^, and mevalonate kinase^33^. However, all of these homologs contain only four strands in the β-sheet, and lack an equivalent for strand β1 of MdcC.

### The subunit interfaces in the MDC hetero-tetramer

Approximately 1100 Å^2^ of the surface area of MdcC is buried in the MDC hetero-tetramer, the majority of which (800 Å^2^) is in the interface with MdcA, while the remainder is in the interface with MdcE. In addition, 400 Å^2^ of the MdcE surface area is buried in an interface with MdcA. On the other hand, MdcD makes only minimal contributions (50 Å^2^) to the surface area burial in MDC outside of its interface with MdcE. The structure therefore indicates that MdcC is an important bridging subunit, mediating the formation of the hetero-tetramer, while the contact between MdcA and MdcD–MdcE (400 Å^2^) is likely too small to form a stable sub-complex.

The interactions in all three interfaces are primarily hydrophilic and ionic in nature. The MdcC–MdcA interface involves the two helices of MdcC contacting several of the loops at the top of the β-sheet of the MdcA AC domain (Fig. 4a). Many of the residues in this interface are well conserved among the MdcA and MdcC homologs (Supplementary Fig. 1). The MdcC–MdcE interface involves two of the long loops at the opposite end of MdcC from Ser25, and strand β12 at the C-terminal edge of the large β-sheet of MdcE (Fig. 4b). The MdcA–MdcE interface involves helix α20 (residues 501–508) just prior to the last strand (β18) of the central β-sheet of the MdcA AC domain and the C-terminal extension of MdcE (residues 241–266, including helix α8A) (Fig. 4c). The MdcA AN domain is not directly involved in holoenzyme formation in this structure.

To assess the assembly of the MDC hetero-tetramer in vitro, we purified MdcA, MdcC (from *P. fluorescens*), and MdcD–MdcE separately and characterized their mixtures by gel filtration. We were able to readily form the hetero-tetramer by mixing purified MdcA, MdcC and MdcD–MdcE (Fig. 4d). Consistent with the structure, we failed to observe stable sub-complexes of the hetero-tetramer when mixing only two or three purified subunits together (Fig. 4e). We also tried to co-express MdcA with MdcD–MdcE in *E. coli*, but observed only soluble MdcA, consistent with the structural information that this sub-complex is unstable.

We also introduced mutations in the interface to test their effects on hetero-tetramer formation and to assess the structural observations. The mutations include a deletion at the C-terminus of MdcA (residues 519–554, with residues 515–525 in the interfaces with MdcC (Fig. 4a) and MdcE (Fig. 4c)), a deletion at the C-terminus of MdcE (residues 242–268, in the interface with MdcA, Fig. 4c), and site-specific mutations at the MdcA–MdcC interface (MdcA F424A, E466A, I490A, and R521A; MdcC R61A, P82A, and T81D-P82E-G83D, Fig. 4a) and at the MdcA–MdcE interface (MdcA R500A, Fig. 4c). The two deletion mutations, the T81D-P82E-G83D triple mutation and the F424A single-site mutation all disrupted the hetero-tetramer, while the other single-site mutations had no appreciable effects on MDC hetero-tetramer formation (Fig. 4f). This is consistent with and supports the structural information in general, and indicates that most single-site mutations are not sufficient to disrupt the interactions in the hetero-tetramer interface.

### The active site of MdcA

**Figure 5:**
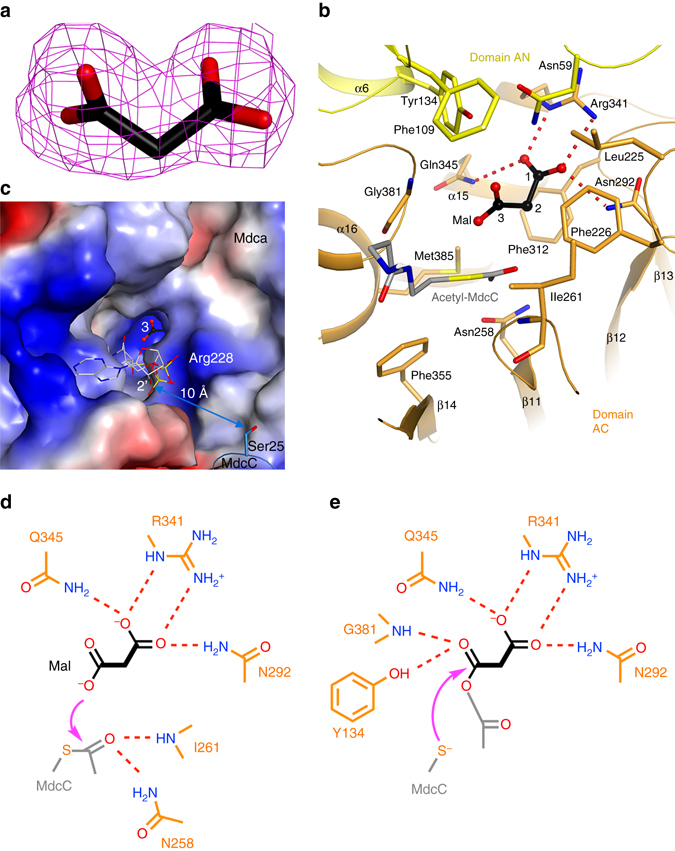
The active site of MdcA. **a** Simulated-annealing omit *F*_o_−*F*_c_ electron density map at 2.2 Å resolution for malonate in the active site of MdcA, contoured at 3*σ*. **b** Interactions between malonate (*black*, labeled Mal, with its carbon atoms numbered) and the MdcA active site. Hydrogen-bonding interactions for the C1 carboxylate of malonate are indicated with dashed lines (*red*). A model for the acetylated prosthetic group of MdcC is shown in *gray*. **c** Molecular surface of the active site region of MdcA, colored by electrostatic potential (*red*: negative, *blue*: positive). Malonate is located at the bottom of a deep, narrow pocket. A model for the acetylated prosthetic group of MdcC is shown in *gray*. **d** Schematic drawing of residues involved in the first step of catalysis by MdcA. The attack of the carboxylate oxygen atom on acetyl-MdcC is indicated by the arrow (*magenta*), and possible residues stabilizing the oxyanion of the transition state are also shown. **e** Schematic drawing of residues involved in the second step of catalysis by MdcA. The attack of the thiolate on the anhydride intermediate is indicated by the arrow (*magenta*)

The active site of MdcA is located at the interface between its AN and AC domains. A malonate molecule from the crystallization solution is bound in the active site, with clearly defined electron density (Fig. 5a). This has provided substantial insights into substrate recognition and catalytic mechanism by this enzyme. One of the carboxylates (C1) of malonate has bidentate ion-pair interactions with Arg341, and its two oxygens are also hydrogen-bonded to the side chains of Asn292 and Gln345, respectively (Fig. 5b). The methylene group (C2) of malonate is situated above the phenyl ring of Phe312. The second carboxylate (C3) is located near Asn59, Tyr134, and Gly381, but is not close enough to have any direct hydrogen-bonding interactions with these residues.

This malonate binding site is located at the bottom of a deep, narrow pocket (Fig. 5c), lined with highly conserved residues among MdcA homologs (Supplementary Fig. 1). This pocket is likely only wide enough to accommodate the other substrate of this enzyme, the acetylated pantothenate portion of the MdcC prosthetic group. We constructed a model for the binding mode of this moiety based on the position of acetyl-CoA in 4-hydroxybutyrate CoA transferase^29^. The acetyl group could be positioned directly next to the malonate in the active site (Fig. 5b). Consistent with MdcA being a class II ACP transferase, there are no acidic residues in the active site that could participate in the catalysis (Fig. 5b), suggesting a direct transfer between malonate and acetyl-MdcC. The C3 carboxylate oxygen of malonate attacks the carbonyl carbon of acetyl-MdcC to produce malonyl-acetyl anhydride and free MdcC, and the main-chain amide of Ile261 and side chain of Asn258 could function as the oxyanion hole to stabilize the transition state of this first reaction (Fig. 5d). The thiolate of MdcC then attacks the malonyl carbonyl in the anhydride to produce malonyl-MdcC and acetate, and the main-chain amide of Gly381 and side chain of Tyr134 could be the oxyanion hole for this reaction (Fig. 5e).

Based on this model, the diphosphate group of the MdcC prosthetic group would be positioned near the side chain of Arg228 (Fig. 5c) as well as the N-terminal end of helix α12 in the MdcA AC domain. The 2′ hydroxyl group of the ribose would be approximately 10 Å from the Ser25 side chain of MdcC (Fig. 5c), suggesting that the current position of MdcC could be catalytically competent and allow its prosthetic group to reach the MdcA active site.

### The active site of MdcD–MdcE

**Figure 6:**
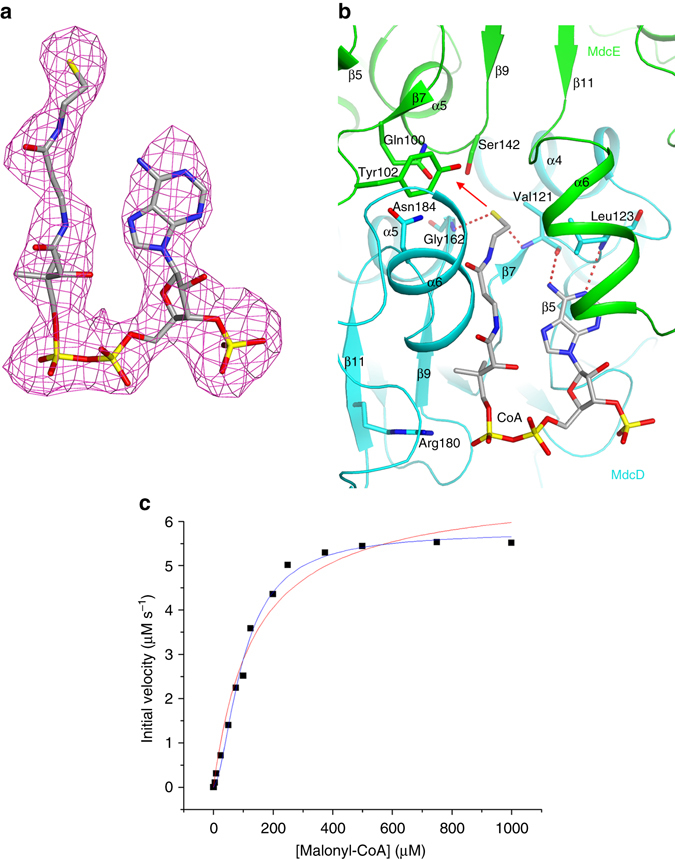
The active site of MdcD–MdcE. **a** Simulated-annealing omit *F*_o_−*F*_c_ electron density map at 3.0 Å resolution for CoA in the active site of MdcD–MdcE, contoured at 3*σ*. **b** Residues in the active site of MdcD–MdcE. Interactions between CoA (*gray*) and MdcD–MdcE are shown. Hydrogen-bonding interactions are indicated with dashed lines (*red*). The possible binding site for the malonyl group is indicated with the red arrow. **c** MdcD–MdcE demonstrates cooperative behavior towards malonyl-CoA. The initial velocity data are fitted to the Hill equation to obtain the kinetic parameters (*blue curve*). The Michael–Menten equation does not produce as good a fit to the data (*red curve*). Data from one representative set of experiments are shown

The active site of MdcD–MdcE is located at their interface, between the small β-sheets of the two subunits. We observed clear electron density for CoA (Fig. 6a), and it primarily interacts with MdcD, with its thiol group located at the center of the interface and having interactions with the main-chain amides of Val121 and Gly162 of MdcD (Fig. 6b). These two amides form the oxyanion hole for this active site, stabilizing the transition state of the decarboxylation reaction. The adenine base is recognized specifically through hydrogen bonds to its N1 and N6 atoms (Fig. 6b). The pyrophosphate group of CoA is located near Arg180 of MdcD, while the 3′ phosphate group of CoA is exposed to the solvent. This is consistent with the fact that the prosthetic group in MdcC is linked to the 2′ hydroxyl of this ribose while the 3′ position is just a hydroxyl (Fig. 1a). The malonyl group likely interacts with the side chains of Gln100, Tyr102, and Ser142 of MdcE (Fig. 6b).

The α6 helices from the two subunits are positioned next to each other (Fig. 6b), forming a lid above the active site such that the active site itself is in a tunnel (Fig. 3b). There are substantial conformational changes for the α6–α6A segment of MdcE in the CoA complex compared to the malonate complex (Supplementary Fig. 6) or the structure of MdcD–MdcE alone. In fact, this helix and the following residues are mostly disordered in the malonate complex and MdcD–MdcE alone. In the CoA complex, helix α6 is positioned close to the adenine base of CoA (Fig. 6b). Conformational differences are also observed for the loop containing residues Val121 and Leu123, which allow these two residues to hydrogen bond and recognize the adenine base of CoA.

### Functional studies based on the structural information

As the MdcC subunit purified from *E. coli* expression lacked the prosthetic group, we were not able to carry out kinetic assays on the overall reaction of MDC. The *A. calcoaceticus* MdcD–MdcE can catalyze the decarboxylation of malonyl-CoA^10^, due to its structural similarity to the prosthetic group (Fig. 1a), and we were able to demonstrate this activity for *P. aeruginosa* MdcD–MdcE as well. Interestingly, *P. aeruginosa* MdcD–MdcE exhibited sigmoidal kinetics toward the malonyl-CoA substrate, with a Hill coefficient of 1.68 ± 0.13 (Fig. 6c), while the *A. calcoaceticus* MdcD–MdcE exhibited hyperbolic kinetics^10^. Enzymes with only a single substrate binding site occasionally demonstrate cooperative behavior, which is oftentimes associated with conformational changes upon substrate binding^34^. The large conformational changes in the MdcD–MdcE active site upon CoA binding (Supplementary Fig. 6) are likely the molecular basis for the observed positive cooperativity. Binding of the malonyl-CoA substrate induces a conformation of the enzyme that facilitates the binding of the next substrate molecule upon product release. The *K*_0.5_ of the reaction is 100 ± 5 μM, while the *k*_cat_ of the enzyme is 2.3 ± 0.1 s^−1^.

We then generated the S142A, Q100E, Y102F, and Q100E/Y102F mutants of MdcE (Fig. 6b) to assess the importance of these residues for catalysis. The S142A and Q100E mutants had a roughly 10-fold lower catalytic activity compared to the wild-type enzyme, while the Y102F mutant had roughly the same activity as wild-type MdcD–MdcE (Supplementary Table 2). Essentially no activity was observed for the Q100E/Y102F mutant. These kinetic studies suggest that Gln100 and Ser142 have important roles for MDC catalysis.

**Figure 7:**
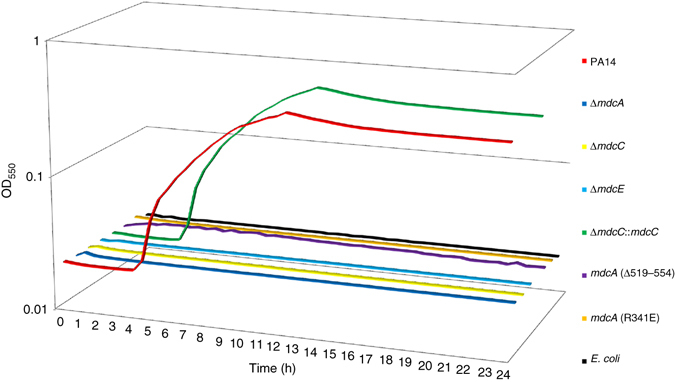
Impact of mutations in MDC holoenzyme subunits on growth with malonate as the sole carbon source. Individual deletion of genes for the MdcA, MdcC, or MdcE subunit in *P. aeruginosa* abolished growth on malonate. Insertion of the coding sequence for the MdcC subunit at the *mdcC* deletion site restored growth. Deletion of MdcA residues 519–554 (required for MDC hetero-tetramer formation) and the R314E mutation in MdcA (involved in recognition of malonate in the active site) also abolished growth. Finally, *Escherichia coli*, which is not able to grow on malonate as a sole carbon source, served as a negative control. Attempts at deleting the MdcD subunit were not successful. Results from one representative experiment from three biological replicates are shown

We next assessed the importance of the MDC subunits for *P. aeruginosa* growth in a defined medium where malonate is the sole carbon source. Wild-type *P. aeruginosa* (strain PA14) grew robustly, consistent with its ability to utilize malonate (Fig. 7). In contrast, wild-type *E. coli* was not able to grow in this medium. As expected, genomic deletion of the *P. aeruginosa* PA14 *mdcA*, *mdcC*, or *mdcE* subunit produced strains that were not able to grow in this medium. Re-introduction of the MdcC subunit into a strain with a genomic deletion of *mdcC* restored growth in this medium.

We then created a mutant that lacked the C-terminal residues 519–554 of the MdcA subunit, which disrupted the MDC hetero-tetramer formation (Fig. 4f). This strain was not able to grow in the defined medium with malonate as the sole carbon source (Fig. 7). We also generated a strain with the R341E point mutation in MdcA. Residue Arg341 is located in the MdcA active site and is crucial for the recognition of the malonate substrate (Fig. 5b). This mutant also failed to grow on malonate. Overall, results from the functional studies are consistent with the structural observations.

## Discussion

Our structures show that the organization of the MdcD–MdcE active site is generally similar to the CT active site of acyl-CoA carboxylases. However, one important difference is that the CT active site binds two substrates, a CoA ester in the β subunit and carboxy-biotin in the α subunit. Therefore, the two subunits of CT are necessary for recognizing its substrates. In contrast, MdcD–MdcE has only one substrate, malonyl-ACP, and our studies show that it primarily interacts with MdcD, with the MdcE subunit contributing to the recognition of the malonyl group. This also explains why MdcE shares much weaker sequence conservation with the α subunit of CT, because they recognize different, unrelated compounds.

The structures have provided substantial insights into the molecular mechanism of substrate recognition and catalysis by MDC, especially MdcA. A collection of residues has been identified that may be important for the activity of this subunit. Unfortunately we were not able to directly test the importance of these residues as we do not have an appropriate assay for MdcA activity. We were able to indirectly confirm the importance of the Arg341 residue that recognizes the malonate carboxylate group by our studies in vivo. For MdcD–MdcE, while we do not know how the malonyl group is recognized by the active site, the structure has identified the oxyanion hole for catalysis, which is conserved in other enzymes with the crotonase fold.

Our kinetic studies showed that *P. aeruginosa* MdcD–MdcE can use malonyl-CoA as a substrate, and therefore can function as an MCD in vitro. The *k*_cat_ of this activity is 14-fold lower and the *k*_cat_/*K*_m_ 38-fold lower than those of human MCD (*k*_cat_ = 33 s^−1^ and *K*_m_ = 38 μM)^7^. Therefore, the MCD activity of MdcD–MdcE appears to be substantially weaker than a canonical MCD enzyme. Further studies will be needed to test whether this activity has any physiological relevance.

The distance between the MdcA and MdcD–MdcE active sites is ~ 60 Å (Fig. 3a). While the MdcC subunit is likely positioned correctly for participating in MdcA catalysis in the current structure, its Ser25 residue is too far from the MdcD–MdcE active site. Therefore, a change in the position of MdcC (and possibly the organization of the MDC hetero-tetramer) is needed for it to participate in the decarboxylation reaction. Further studies are needed to define the structure of this second state of the hetero-tetramer, as well as how MDC switches between the two states during catalysis.

The structure of the acyl-carrier protein in MDC, MdcC, is distinct from that in fatty acid synthase (FAS), and the prosthetic group attached to MdcC is distinct from that in FAS as well. Therefore, our studies have revealed a new acyl carrier, illuminating the natural diversity in these proteins. In fact, results from the DaliLite search indicate that MdcC may have a distinct backbone fold compared to all other known structures. On the other hand, MdcC is likely similar to the acyl carrier in the MAD and citrate lyase, and therefore the structural information obtained for this subunit here may also be relevant for these other enzymes.

The domains of MdcA have strong structural similarity to those of CoA and ACP transferases, confirming that MdcA is a member of this superfamily. On the other hand, the relative positioning of the two domains in MdcA has substantial difference to that in the other family members, indicating some degree of variability. This might be important for the remarkable diversity in the substrates that are recognized by these enzymes.

Various pseudomonads can utilize malonate as a sole carbon source for growth. Our functional studies indicate that removal of MDC subunits, a point mutation in the MdcA active site, or a C-terminal truncation of MdcA that abolishes hetero-tetramer formation all prevent growth on this substrate. These results are consistent with and support our structural observations, and demonstrate the importance of the MDC holoenzyme for malonate utilization.

## Methods

### Protein expression and purification

Full-length genes of *mdcD* and *mdcE* were amplified by PCR (Supplementary Table 3) from the *P. aeruginosa* genomic DNA (ATCC) and cloned into the vector pCDFDuet (Novagen). N-terminal His-tagged MdcE was cloned into MCS1 and untagged MdcD was cloned into MCS2. The identification of successful clones was verified by DNA sequencing.

For the holoenzyme, full-length genes of *mdcA* and *mdcC* were amplified by PCR from the *P. aeruginosa* and *P. fluorescens* genomic DNA (ATCC), respectively. The two genes were cloned bicistronically into the vector pET28a, with no affinity tags on either protein.

The MdcD–MdcE complex was over-expressed in *E. coli* BL21Star (DE3) strain (Novagen). Cells in 6 l of LB were induced at OD_600_ of 0.6 by 1 mM isopropyl β-d-1-thiogalactopyranoside (IPTG) and grown for 16 h at 18 °C. Cells were harvested by centrifugation, resuspended in a lysis buffer containing 20 mM Tris (pH 7.5), 500 mM NaCl and 50 mM imidazole and lysed by sonication. The cell lysate was centrifuged at 25,000×*g* for 30 min at 4 °C, and the supernatant was incubated with nickel beads (Qiagen). After 1 h, beads were transferred to a plastic gravity flow column (Bio-Rad) and washed with lysis buffer and eluted using a buffer containing 20 mM Tris (pH 7.5), 500 mM NaCl, and 500 mM imidazole. The eluate was directly loaded onto a gel filtration column (Sephacryl S-300; GE Healthcare) equilibrated in a buffer containing 20 mM Tris (pH 7.5), 300 mM NaCl, and 1 mM DTT. The peak fractions were collected and concentrated to 20 mg ml^−1^, frozen in liquid nitrogen and stored at −80 °C.

The selenomethionyl MdcD–MdcE complex was prepared by growing the auxotrophic *E. coli* strain B834 (DE3) in LeMaster medium^35^. The protein was purified following the same protocol as that for the native protein except that 10 mM DTT was used in the buffer during gel filtration.

The MDC hetero-tetramer was over-expressed by co-transforming *E. coli* BL21 Rosetta cells (Novagen) with pET28a-*mdcAC* and pCDFDuet-*mdcDE*. Cells were induced at OD_600_ of 0.6 by 1 mM IPTG and grown for 16 h at 18 °C. The hetero-tetramer was purified following the same protocol as that for the MdcD–MdcE complex. The peak fractions were collected and concentrated to 15 mg ml^−1^.

### Protein crystallization

Initial screening was performed using sparse matrix conditions with the aid of a Mosquito robot. The MdcD–MdcE complex was at 10 mg ml^−1^ concentration. Crystals were obtained at 20 °C with the sitting-drop vapor diffusion method, using a reservoir solution containing 0.1 M Bis—Tris (pH 5.8), 18% (w/v) PEG 3350, and 0.2 M ammonium acetate.

The MDC hetero-tetramer was at 15 mg ml^−1^ concentration for crystallization. Needle-shaped crystals were obtained at 20 °C with the sitting-drop vapor diffusion method, using a reservoir solution containing 20% (w/v) PEG 3350 and 8% Tacsimate. Two rounds of seeding, performed using a Seed Bead (Hampton), with incubation at 4 °C yielded single crystals suitable for data collection. Heavy atom soaks were performed using K_3_Au(CN)_6_ at a final concentration of 1 mM at 4 °C for 16 h.

For soaking with malonyl-CoA, crystals of the hetero-tetramer were transferred into a fresh sitting drop containing mother liquor (8% Tacsimate pH 8.0, 15% (w/v) PEG 3350), and malonyl-CoA was added to the drop to a final concentration of 0.5 mM. The crystal was incubated for 16 h at 4 °C before being harvested and cryo-protected in the mother liquor containing 15% (v/v) PEG 200.

### Data collection and structure determination

For the MdcD–MdcE complex, a single-wavelength anomalous diffraction dataset at 1.8 Å resolution was collected at 100 K at the peak absorption wavelength of selenium (0.9790 Å) at the X25 beamline of the National Synchrotron Light Source, using a Pilatus 6M detector. The diffraction images were processed using the HKL2000 package^36^. The structure was solved using the program PHENIX^37^. Manual model rebuilding was performed using Coot^38^, and the structure refinement was carried out using PHENIX.

The structure of the MDC hetero-tetramer was determined by the single isomorphous replacement method. A native dataset was collected at 3.0 Å resolution at the NE-CAT 24-IDC beamline at the Advanced Photon Source. A single-wavelength anomalous diffraction dataset at 3.5 Å resolution was collected at the peak absorption wavelength of gold (1.0402 Å) on the crystal soaked with K_3_Au(CN)_6_. Four gold sites were located with isomorphous difference Patterson map^39, 40^ as well as isomorphous difference electron density map using phases calculated from the MdcD–MdcE model, which was placed into the holoenzyme crystal by the molecular replacement method using the program COMO^41^. The reflections were phased using the program PHENIX^37^. Phase improvement, with two-fold non-crystallographic symmetry averaging, was carried out with Resolve^42^. The models for MdcA and MdcC were built manually with Coot. The gold compound is bound to Cys22 of MdcC and Cys230 of MdcE, respectively.

A new native crystal of the hetero-tetramer was produced in the presence of 20 mM 18-crown-6 crown ether as an additive^43^, which diffracted to 2.2 Å resolution. The structure was solved by the molecular replacement method with the program COMO, and structure refinement was carried out with CNS^44^ and PHENIX. No electron density for the crown ether was observed in the crystal.

The diffraction data on the CoA complex was processed with the program XDS^45^. The structure of the complex was determined by the molecular replacement method with the program COMO, using the structure of one MDC hetero-tetramer as the search model. NCS restraints among the hetero-tetramers were used throughout the refinement.

### Model of acetyl-CoA in MdcA active site

The structure of the AC domain of MdcA was superposed with the equivalent domain in the structure of 4-hydroxybutyrate CoA transferase in complex with acetyl-CoA (PDB entry 4N8I)^29^. This brings the acetyl group close to the malonate molecule in the MdcA active site. The torsion angle of the thioester bond between the acetyl and the thiol groups was changed by 180° to make the carbonyl oxygen pointed toward the putative oxyanion holes, followed by manual adjustments to avoid clashes between the acetyl-CoA and MdcA.

### In vitro reconstitution of the MDC holoenzyme

Full-length, N-terminally His-tagged MdcA from *P. aeruginosa* was over-expressed in *E. coli* BL21(DE3) Rosetta cells using the vector pCDFDuet (Novagen) with *mdcABC* cloned into MCS1 and *mdcDE* cloned into MCS2. The genes were PCR amplified directly from the operon from *P. aeruginosa* genomic DNA. Only MdcA was soluble and the other four proteins were not observed using this expression construct. In addition, co-expression of MdcA with MdcD–MdcE also yielded soluble MdcA, but no MdcD–MdcE. In contrast, expressing MdcA alone, in pET28a or pET26b, produced only insoluble protein.

Full-length, C-terminally His-tagged MdcC from *P. fluorescens* was over-expressed in *E. coli* BL21Star (DE3) cells. The gene was amplified by PCR from *P. fluorescens* genomic DNA and cloned into pET26b (Novagen).

Large-scale protein expression and purification were carried out for MdcA, MdcC and the MdcDE dimer, as described above, and the proteins were concentrated to 6, 3 and 20 mg ml^−1^ respectively. Both MdcA and MdcC were found to be unstable unless the gel filtration buffer was supplemented with 50 mM arginine and 10% (v/v) glycerol.

Complex reconstitution experiments were carried out using gel filtration with a Superose 12 column (GE Healthcare) with a buffer consisting of 20 mM Tris (pH 7.5), 300 mM NaCl, 1 mM DTT, 10% (v/v) glycerol, and 50 mM arginine. Each combination of proteins was incubated on ice for 1 h to allow complex formation and then loaded onto the column. The substrate malonate was not included in the buffer. Binding of malonate does not cause a major change in the structure of MdcA (as evidenced by comparing the structures of the malonate and CoA complexes), and it is unlikely to change the 1:1:1:1 stoichiometry and the gel filtration behavior of the holoenzyme.

### Mutagenesis

The mutants were made with the QuikChange kit (Stratagene) and verified by sequencing. The mutant proteins were expressed and purified following the same protocol as that for the wild-type protein.

### Kinetic assays on MdcD–MdcE

The decarboxylase activity of wild-type and mutant MdcD–MdcE complexes toward malonyl-CoA was determined using a coupled-enzyme assay, converting acetyl-CoA production to NAD^+^ reduction^46^. The reaction contained 2.5 μM MdcD–MdcE, 6 mM l-malate, 0.5 mM NAD^+^, 1 U malate dehydrogenase (Sigma), 0.2 U citrate synthase (Sigma), 20 mM Tris (pH 8.0), and various concentrations of malonly-CoA. The production of NADH was monitored by absorbance at 340 nm.

### Generation of mutant *Pseudomonas aeruginosa* strains

Strains and primers used to generate *P. aeruginosa* mutants for this study are listed in Supplementary Tables 4 and 5. Mutants were created in the *P. aeruginosa* strain UCBPP-PA14^47^ using homologous recombination^48^. Constructs for generating deletions, complementations, and point mutations were made using the yeast gap repair method^49^ by employing the allelic-replacement vector pMQ30 and the yeast strain InvSc1 (Invitrogen). For deletion of a specific gene, ~ 1 kb flanking regions plus ~ 100 bp of each end of the coding region were amplified and cloned into pMQ30. For complementation of Δ*mdcC*, the full coding region plus ~ 1 kb flanking regions were amplified and cloned into pMQ30. To generate individual point mutants, a portion of the gene of interest was amplified using a primer pair that added the point mutation in an overhang. Resultant plasmids were propagated in *E. coli* UQ950, transformed into the mating strain *E. coli* BW29427, and moved into PA14 by conjugation. Recombinants were plated onto selective media containing 100 μg ml^−1^ gentamicin. Candidate mutant or complementation strains were identified by counter-selection on LB agar containing 10% (w/v) sucrose and no NaCl. Verification of the final clones was obtained via PCR or sequencing.

### Malonate growth assay

All strains were grown for 16 h in LB broth at 37 °C. Cells were centrifuged and re-suspended in MOPS (4-morpholinepropanesulfonic acid) defined medium supplemented with malonate (50 mM MOPS (pH 7.2), 43 mM NH_4_Cl, 2.2 mM KH_2_PO_4_, 1 mM MgSO_4_·7H_2_O, 3.57 mM FeSO_4_·7H_2_O, 20 mM sodium malonate). Re-suspended cells were diluted into fresh MOPS medium at a starting OD_550_ of 0.1. Experiments were performed in triplicates using a 96-well plate reader (BioTek Synergy 4) set to incubate at 37 °C and shake continuously, and OD_550_ readings were taken every 30 min for 24 h.

### Data availability

Coordinates and structure factors have been deposited in the PDB with accession codes 5VIP, 5VIT, and 5VJ1. Other data supporting the findings of the manuscript are available from the corresponding author upon reasonable request.

## Electronic supplementary material

Supplementary Information
