# Supplementary material for: Crystal structure of a Pseudomonas malonate decarboxylase holoenzyme hetero-tetramer
Source: Nat Commun. 2017 Jul 31;8:160. doi: 10.1038/s41467-017-00233-z (PMC5534430; doi:10.1038/s41467-017-00233-z)
Supplement: Supplementary file 1 — Supplementary Information [file 41467_2017_233_MOESM1_ESM.pdf]

File name: Supplementary Information
Description: Supplementary figures and supplementary tables.

Supplementary Table 1

Structural homologs of domains AN and AC of MdcA

**Structural homologs**	**Family**	***Z* score**	**Sequence identity (%)**
**Domain AN**			
succinyl-CoA:3-ketoacid CoA transferase (SCOT, and PDB entries 3CDK, 1O9L, 3DLX, 3RRL)	I	21	20
acetyl-CoA transferase	I	21	17
acyl-CoA transferase YdiF	I	19	20
gluconate-CoA transferase	I	18	20
succinyl-CoA:acetate CoA transferase	I	16	18
citrate lyase α subunit (citrate-ACP transferase, PDB entries 1XR4 and 2HJ0)	II	18	16
**Domain AC**			
4-hydroxybutyrate CoA transferase (and PDB entry 2OAS)	I	18	17
succinyl-CoA:acetate CoA transferase	I	17	13
succinyl-CoA:3-ketoacid CoA transferase	I	12	14
citrate lyase α subunit (PDB entries 2HJ0 and 1XR4)	II	17	16

Supplementary Table 2

Catalytic activities of MdcE mutants

**Protein**	**Initial velocity**[1]
Wild-type	2.02×10^{-3} (1)
Q100E	2.58×10^{-4} (8)
Y102F	1.89×10^{-3} (1)
S142A	1.81×10^{-4} (11)
Q100E-Y102F	7.11×10^{-6} (280)

[1] The reaction contained 125 μM malonyl-CoA as the substrate. The values in parentheses are the fold change compared to the activity of the wild-type enzyme.

Supplementary Table 3

Primers used for making MDC bacterial expression constructs

**Gene[1]**	**UniProt entry ID for protein**	**Primers**
MdcA	A6UY03	Forward: GCGCCATGGCGACACCGATCTCTCC<br>Reverse: GCGGAATTCTTACCAGCTCCTGAAACG
MdcC	Q3K5B7	Forward: GCGCCATGGAAACCTTGTCC<br>Reverse: GCCCTCGAGTTAGTCATGGCCGATCTCC
MdcD	A6UY06	Forward: GCCCATATGACTGACGTCGCCCGCCTGC<br>Reverse: GCCCTCGAGTTATGTTGCATCTCCTTGGCCCAGC
MdcE	A6UY07	Forward: GCCGAATTCGAGCCAACCGTTCGCTTCCCGTGG<br>Reverse: GCCAAGCTTTTACCACTGCTCGCGCAGCAG

[1]MdcA, MdcD and MdcE are from *P. aeruginosa*, and MdcC from *P. fluorescens*.

Supplementary Table 4

Bacterial and fungal strains used in this study

Strain	Number	Relevant characteristics	Source
Pseudomonas aeruginosa			
UCBPP-PA14		Clinical isolate UCBPP-PA14	Ref. 1
PA14 Δ*mdcA*	LD2504	PA14 with deletion in PA14_02550	this study
PA14 Δ*mdcC*	LD2538	PA14 with deletion in PA14_02570	this study
PA14 Δ*mdcE*	LD2510	PA14 with deletion in PA14_02590	this study
PA14 Δ*mdcC::mdcC*	LD2674	PA14 Δ*mdcC* with *mdcC* (PA14_02570) reinserted at site of deletion	this study
PA14 *mdcA R341E*	LD2741	PA14 containing R314E mutation in the active site of MdcA	this study
PA14 *mdcA Δ519-554*	LD2752	PA14 containing a deletion in the coding sequence for MdcA from residues 519-554	this study
Escherichia coli			
UQ950		*E. coli* DH5 λ(pir) strain for cloning. F$^-$ Δ(*argF-lac*)*169* ϕ80d*lacZ58*(ΔM15) *glnV44(*AS) *rfbD1 gyrA96*(Nal^R) *recA1 endA1 spoT1 thi-1 hsdR17 deoR* λpir$^+$	D. Lies, Caltech
BW29427		Donor strain for conjugation. *thrB1004 pro thi rpsL hsdS lacZ* ΔM15 *RP4-1360* Δ(*araBAD*)*567* Δ*dapA1314::*[*erm pir*(wt)]	W. Metcalf, University of Illinois
Saccharomyces cerevisiae			
InvSc1		*MAT***a***/MAT*α *leu2/leu2 trp1-289/trp1-289 ura3-52/ura3-52 his3-Δ1/his3-Δ1*	Invitrogen

1 Rahme, L. G. *et al*. Common virulence factors for bacterial pathogenicity in plants and animals. *Science* **268**, 1899-1902 (1995).

Supplementary Table 5

Primers used in this study

ΔmdcA	
ΔmdcA yeast-1	ccaggcaaattctgttttatcagaccgcttctgcgttctgaCTTGCGCTGGATCAACTCG
ΔmdcA yeast-2	agctcggcgatgctcttcgcCTTCTGGTTGTTGCCTTC
ΔmdcA yeast-3	gaaggcaacaaccagaagGCGAAGAGCATCGCCGAGCT
ΔmdcA yeast-4	ggaattgtgagcggataacaatttcacacaggaaacagctCATCTCAGAGACTCCCAGCC
ΔmdcC	
ΔmdcC yeast-1	ccaggcaaattctgttttatcagaccgcttctgcgttctgaGAAGGCGTGAACACCCATC
ΔmdcC yeast-2	gggagtggcgccgaaatcGGCGGGAAATTCGAAGGTCAG
ΔmdcC yeast-3	ctgaccttcgaatttcccgccGATTTCGGCGCCACTCCC
ΔmdcC yeast-4	ggaattgtgagcggataacaatttcacacaggaaacagctAGTCCGAGGTTGGCTTCC
ΔmdcE	
ΔmdcE yeast-1	ccaggcaaattctgttttatcagaccgcttctgcgttctgatGCTGGCCATGACTGACGT
ΔmdcE yeast-2	acgcgtagctgtcgatgtcGCGCAACGAAGCAGGATC
ΔmdcE yeast-3	gatcctgcttcgttgcgcGACATCGACAGCTACGCGT
ΔmdcE yeast-4	ggaattgtgagcggataacaatttcacacaggaaacagctCGATCAGCAGGCACAACT
ΔmdcC::mdcC	
ΔmdcC yeast-1	ccaggcaaattctgttttatcagaccgcttctgcgttctgaGAAGGCGTGAACACCCATC
ΔmdcC yeast-4	ggaattgtgagcggataacaatttcacacaggaaacagctAGTCCGAGGTTGGCTTCC
mdcA R341E	
mdcA yeast-1	ccaggcaaattctgttttatcagaccgcttctgcgttctgatCCCGGCTTGATTGAACAG
mdcA yeast-2	GGCCAGTTGGCAGAACATctcATTGGAACGCAGCGAGCC
mdcA yeast-3	GGCTCGCTGCGTTCCAATgagATGTTCTGCCAACTGGCC
mdcA yeast-4	ggaattgtgagcggataacaatttcacacaggaaacagctATGGGTGTTCACGCCTTC
mdcA Δ519-554	
mdcA del yeast-1	ccaggcaaattctgttttatcagaccgcttctgcgttctgatGTGCTGATGGCGATGATG
mdcA del yeast-2	tcgcgatggcgttcatcaGCCGAGGTCCTCGGGCAG
mdcA del yeast-3	ctgcccgaggacctcggcTGATGAACGCCATCGCGA
mdcA del yeast-4	ggaattgtgagcggataacaatttcacacaggaaacagctCATCTCAGAGACTCCCAGCC

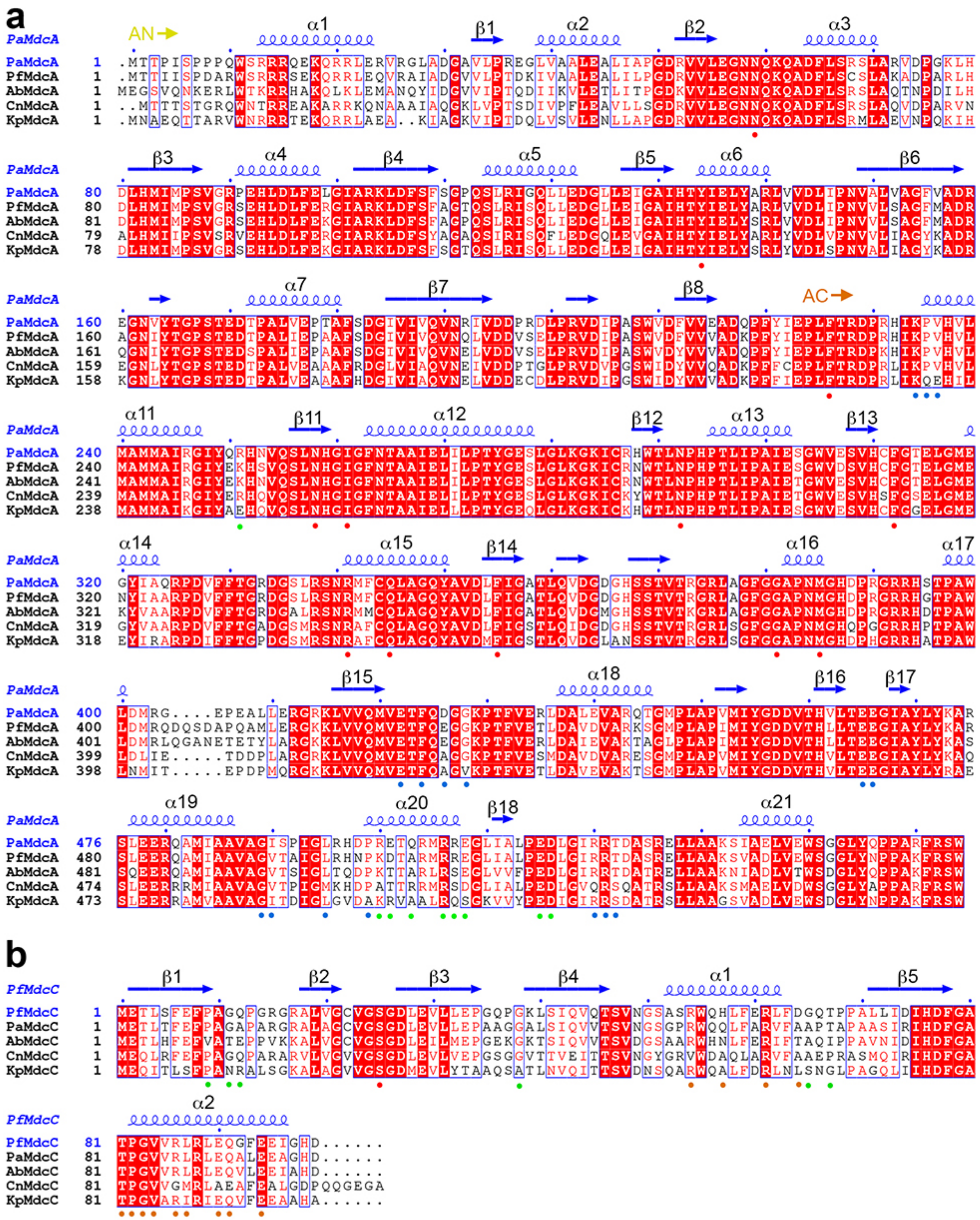
a
PaMdcA
PfMdcA
AbMdcA
CnMdcA
KpMdcA
b
PfMdcC
PaMdcC
AbMdcC
CnMdcC
KpMdcC

Supplementary Fig. 1. Sequence alignment of MdcA and MdcC subunits. (**a**). Sequence alignment of MdcA. The observed secondary structure elements are shown in blue above the sequence and labeled. Residues in the active site are indicated with the red dots. MdcA residues in contact with MdcC are indicated with the blue dots, and those in contact with MdcE with the green dots. The domain boundaries are indicated with the arrowheads and labeled. Pa: *Pseudomonas aeruginosa*, Pf: *Pseudomonas fluorescens*, Ab: *Acinetobacter baumannii*, Cn: *Cupriavidus necator*, Kp: *Klebsiella pneumoniae*. (**b**). Sequence alignment of MdcC. MdcC residues in contact with MdcA are indicated with the orange dots. Modified from an output from ESPript [2]

2 Gouet, P., Courcelle, E., Stuart, D. I. & Metoz, F. ESPript: analysis of multiple sequence alignments in PostScript. *Bioinformatics* **15**, 305-308 (1999).

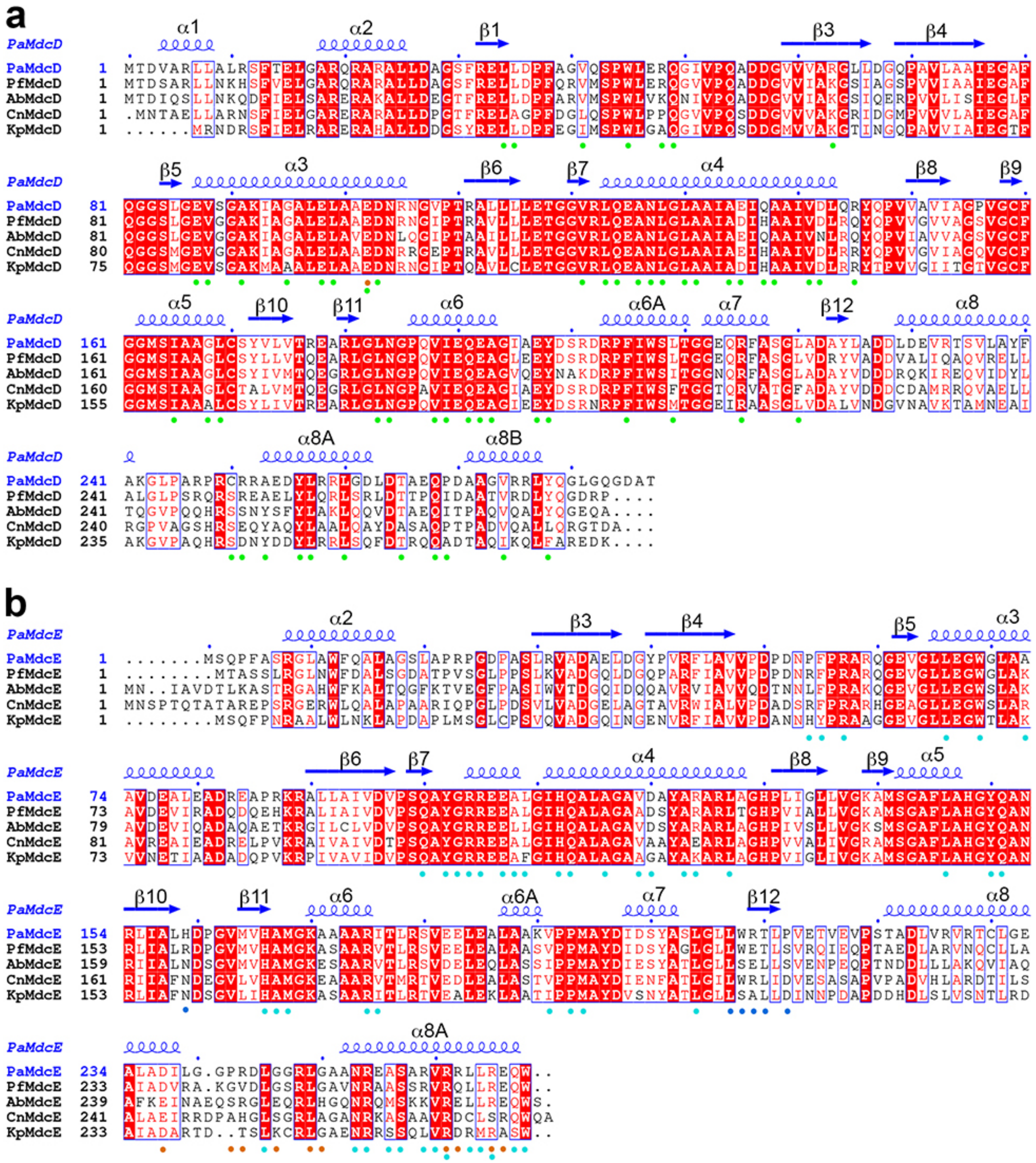

Supplementary Fig. 2. Sequence alignment of MdcD and MdcE subunits. (**a**). Sequence alignment of MdcD. (**b**). Sequence alignment of MdcE.

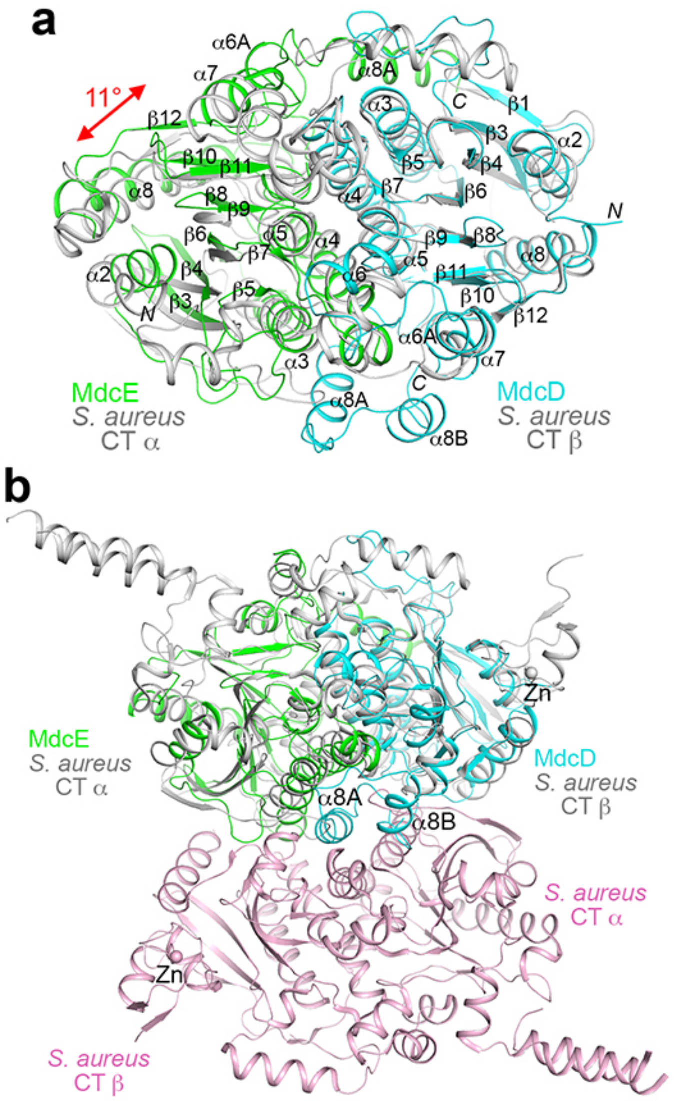

Supplementary Fig. 3. Structural comparison between MdcD-MdcE with ACC CT. (**a**) Overlay of the structure of the MdcD-MdcE complex (in color) with that of the CT αβ hetero-dimer of *S. aureus* ACC (gray). The overlay is based on MdcD, and an 11° difference in the orientation of MdcE relative to the CT α subunit is observed. (**b**) Overlay of the structure of the MdcD-MdcE complex (cyan and green) with that of a CT αβ hetero-dimer in the $\alpha_2\beta_2$ hetero-tetramer of *S. aureus* ACC (gray and pink). The α8A and α8B helices at the C-terminal end of MdcD are incompatible with the interface between the two CT αβ hetero-dimers.

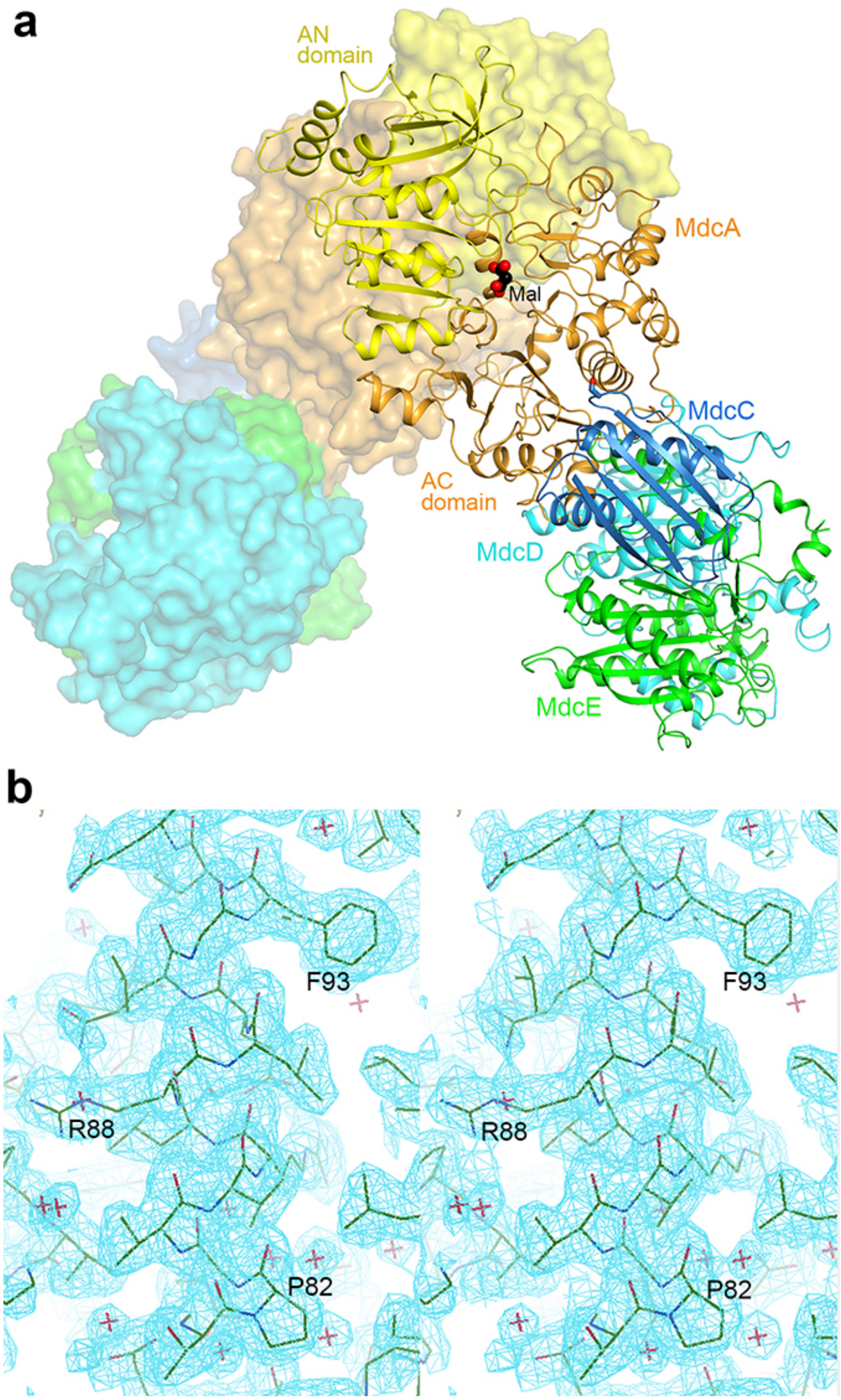

Supplementary Fig. 4. (**a**) A dimer of the MDC in the crystal. One MDC hetero-tetramer is shown as ribbons, while the other as a surface. The dimer interface is mediated solely by MdcA. MDC is a hetero-tetramer in solution. (**b**) Stereo view of $2F_o–F_c$ electron density at 2.2 Å resolution for a helix in MdcA, contoured at 1σ.

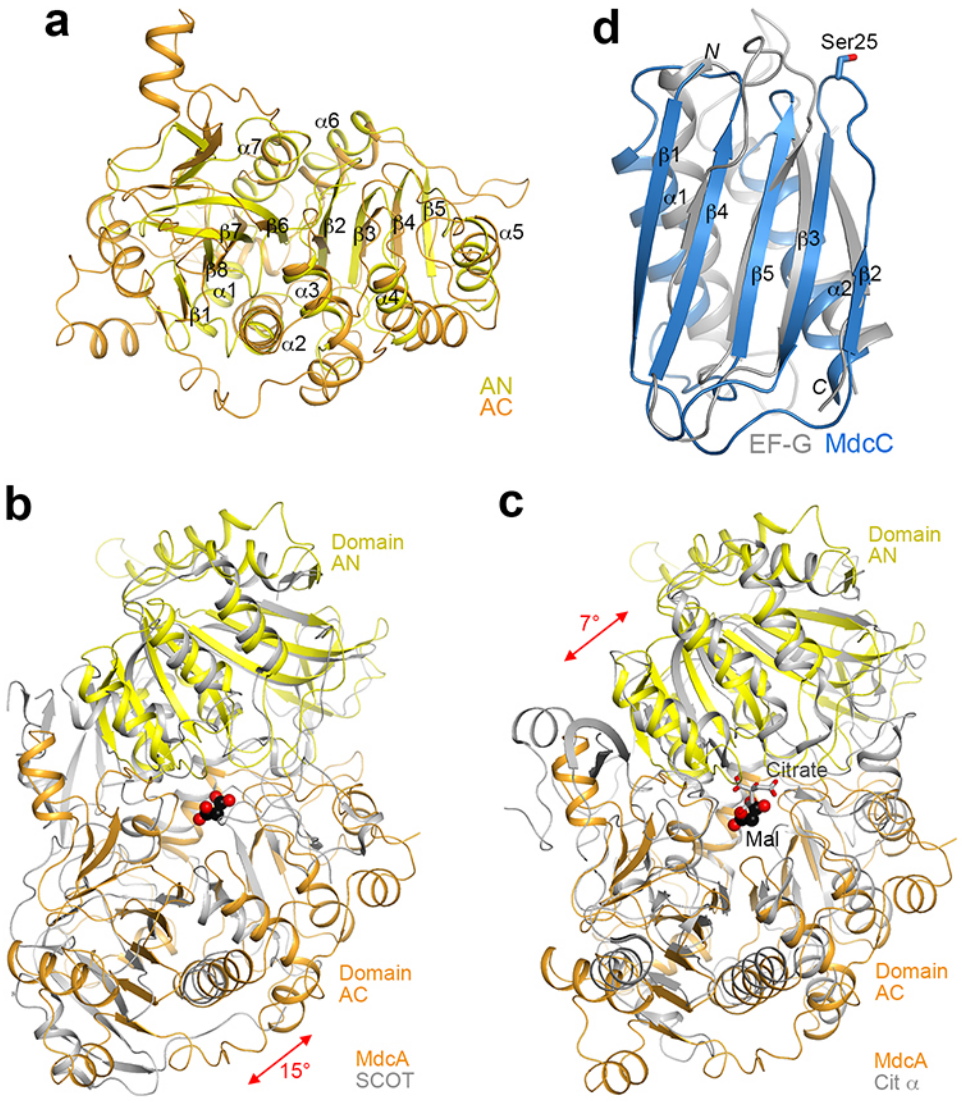

Supplementary Fig. 5. Structural homologs of MdcA and MdcC. (**a**) Overlay of the structures of the MdcA AN (yellow) and AC (orange) domains. (**b**) Overlay of the structure of MdcA (in color) with that of succinyl-CoA:3-ketoacid CoA transferase (SCOT, *Z* score 21, 20% sequence identity, gray). The overlay is based on the AN domain, and a 15° difference in the orientation of the AC domain relative to the equivalent in SCOT is observed. Malonate is shown as a ball-and-stick model (black). (**c**) Overlay of the structure of MdcA (in color) with that of citrate lyase α subunit (Cit α, *Z* score 18, 16% identity). The overlay is based on the AC domain, and a 7° difference in the orientation of the AN domain relative to the equivalent in citrate lyase is observed. Citrate is shown as a stick model (gray). (**d**) Overlay of the structure of MdcC (in color) with that of domain IV of elongation factor G (EF-G, gray).

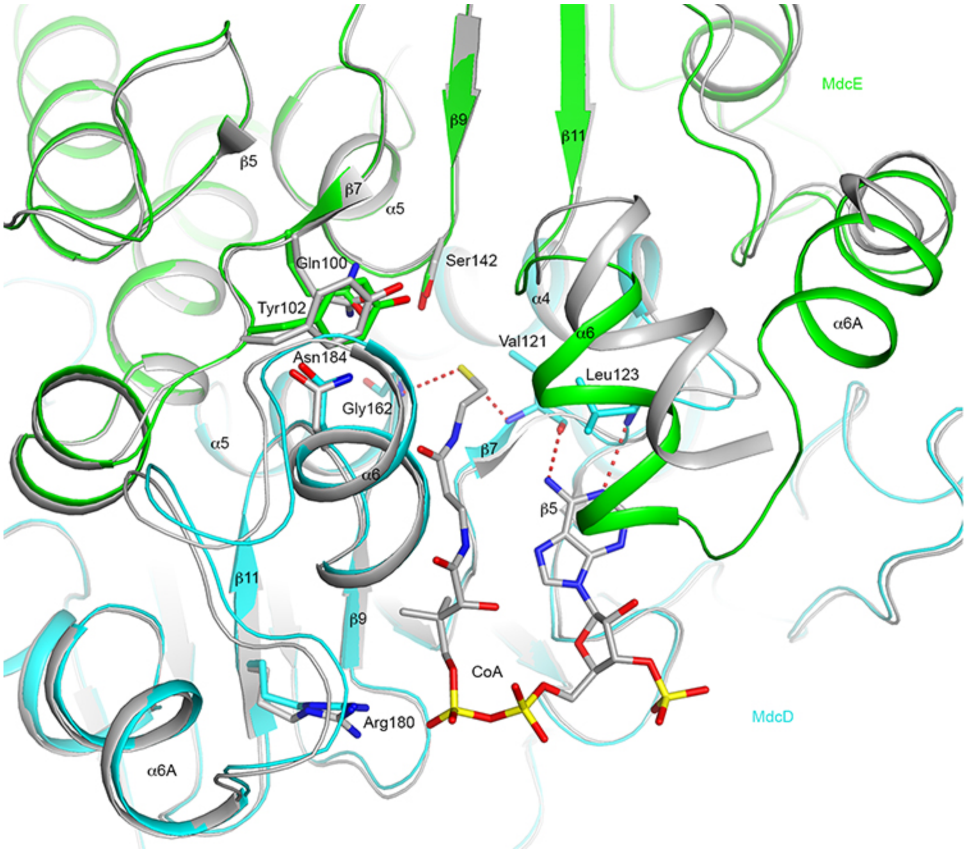

Supplementary Fig. 6. Conformational changes in MdcD-MdcE during catalysis. The active site region of MdcD-MdcE in the CoA complex (in color, with the CoA in gray) is overlaid with that in the malonate complex (gray). Differences are seen for the α6-α6A segment of MdcE, the loop containing residues Val121 and Leu123, and the α6-α6A segment of MdcD.
